# Genome-wide identification and functional characterization of alpha-amylase genes in *Litopenaeus vannamei*

**DOI:** 10.1371/journal.pone.0338707

**Published:** 2026-01-13

**Authors:** Jingxuan Liang, Hao Yang, Guiling Tan, Ting Chen, Lvping Zhang, Wenjie Pan, Jiayue Yin, Bo Ma, Jianfeng Xu, Jiquan Zhang, Peng Luo, Peiming Zheng, Chaoqun Hu, Chunhua Ren

**Affiliations:** 1 State Key Laboratory of Breeding Biotechnology and Sustainable Aquaculture, Laboratory of Tropical Marine Bio-resources and Ecology, Guangdong Provincial Key Laboratory of Applied Marine Biology, South China Sea Institute of Oceanology, Chinese Academy of Sciences, Guangzhou, China; 2 University of Chinese Academy of Sciences, Beijing, China; 3 Laboratory of Zoological Systematics and Application of Hebei Province, College of Life Sciences, Hebei University, Baoding, China; 4 Guangdong Jinyang Biotechnology Co., Ltd, Maoming, China; Universiti Malaysia Kelantan, MALAYSIA

## Abstract

Alpha-amylase is a key enzyme involved in carbohydrate hydrolysis and food digestion in animals. However, its molecular characteristics and functions remain poorly understood in the economically important Pacific white shrimp, *Litopenaeus vannamei*. Through comparative genomic analysis, this study revealed that the α-amylase (*Amy*) gene family has undergone an expansion in arthropods, particularly crustaceans, while retaining highly conserved catalytic domains. Six *Amy* genes were identified in *L. vannamei*. Their expression patterns across tissues and developmental stages revealed predominant transcription in the hepatopancreas, with significant upregulation during periods of high energy demand, such as zoeal feeding and pre-molting. Among these genes, *Lv-Amy* (XP_027225605.1) exhibited the highest expression level. Quantitative real-time PCR (qPCR) and fluorescence *in situ* hybridization (F*IS*H) further characterized the tissue distribution and cellular localization of *Lv-Amy*. Structurally, *Lv*-Amy displays a typical (β/α)₈ TIM barrel conformation and two conserved calcium-binding sites. The recombinant *Lv*-Amy protein was produced in an *E. coli* expression system and its enzymatic properties were characterized. Maximal activity of the recombinant *Lv*-Amy protein was observed at pH 7.5 and 25°C, while activity remained above 50% of the maximum within pH 7.0–8.0 and 20–45°C, indicating that *Lv-*Amy can function efficiently under environmental conditions characteristic of the tropical marine habitat of *L. vannamei*. In summary, this study provides new insights into the molecular characteristics and functions of α-amylase in *L. vannamei*, suggesting that *Lv-Amy* plays an important role in digestion and offering a reference of stage-specific nutritional formulation for this species.

## Introduction

Digestive enzymes in animals with different feeding habits, such as, herbivores, omnivores, and carnivores, exhibit distinct characteristics. In general, herbivores display higher carbohydrate-active enzymes (CAZymes) activity, whereas carnivores possess higher protease and lipase activity [1,2]. Most crustaceans, including *L. vannamei*, are omnivorous. Their diverse nutritional sources confer the ability to degrade and transform a wide range of nutrient substrates [3], a capability that primarily depends on the adaptive regulation of digestive enzymes in response to different dietary compositions [4]. Digestive enzymes not only reflect their feeding patterns and nutritional status in crustaceans but are also closely linked to their circadian rhythms and molting cycles [5]. Furthermore, while amylase activity is typically higher in omnivorous vertebrates compared to other digestive enzymes [6], its pattern in omnivorous crustaceans is less understood.

Amylase is a class of enzymes that catalyze the hydrolysis of starch and glycogen and are widely distributed across living organisms. The first amylase was identified in 1811, and by 1930, amylase was classified into α- and β-type based on their reaction products [7]. Currently, they are further divided into α-amylase (EC 3.2.1.1), β-amylase (EC 3.2.1.2) and γ-amylase (EC 3.2.1.3). Within the sequence-based classification system of CAZymes, α-amylase is one of the most prevalent glycoside hydrolases (GHs) [8]. It is a calcium-dependent metalloenzyme with broad substrate preference and product specificity, catalyzing the hydrolysis of α-1,4-glycosidic bonds in the linear chains of starch and glycogen to generate maltose, maltotriose and residual branched maltodextrins [9,10]. To date, amylases have been identified in various organisms, including vertebrates [11], invertebrates [12], plants [13], and microorganisms [14]. Due to its crucial role in carbohydrate metabolism and broad taxonomic distribution, the studies on α-amylase may offer valuable insights into the digestive physiology of crustaceans.

The Pacific white shrimp, *Litopenaeus vannamei*, is one of the most extensively farmed crustaceans worldwide [15]. Although three α-amylase (*Amy*) genes have been identified in *L. vannamei* [16], comprehensive studies regarding their evolutionary relationships, expression patterns across developmental stages, and enzymatic properties remain limited. Moreover, compared with other arthropods, such as insects, research on α-amylase in crustaceans is still scarce [17]. In this study, we performed a systematic analysis of the *Amy* gene family in *L. vannamei*. Phylogenetic approaches were employed to investigate their evolutionary relationships within arthropods, while bioinformatic methods were used to predict their domain compositions. By integrating transcriptomic data, we further characterized the expression patterns of the *Amy* gene family across tissues, developmental stages, and molting cycles in *L. vannamei*. Based on this, we identified a predominant *Amy* gene (*Lv-Amy*) at the transcriptional level, and conducted in-depth analyses of its tissue distribution, cellular localization, and the enzymatic properties of its recombinant protein. This study intends to systematically elucidate the key roles of α-amylase in digestive physiology and energy metabolism in *L. vannamei*, to advance understanding of the molecular mechanisms underlying carbohydrate utilization in shrimp, and to provide a reference of stage-specific nutritional formulation for this species.

## Materials and methods

### Sampe collection and ethical statement

All the adult *L. vannamei* were obtained from the Jinyang Shrimp Culture Center (Maoming, Guangdong, China) where they were stably farmed in artificial seawater (30 ppt, pH 8.2) at 28°C. The shrimps were dissected on ice, and the tissues were quickly collected and immediately placed in liquid nitrogen for temporary storage. A total of ten tissues were collected, including hepatopancreas (Hp), stomach (St), intestine (In), heart (Ht), gill (Gi), eyestalk (Es), brain (Br), thoracic nerve (TN), abdominal nerve (AN) and muscle (Ms). In addition, embryos and larval stages were generated from parent shrimps maintained under identical conditions and collected at the following stages: early fertilized egg (pre-E), late fertilized egg (post-E), nauplius stages (N1 − N6), zoea stages (Z1 − Z3), mysis stages (M1 − M3) and postlarvae (Pl). All samples were stored at −80°C until further used for further experiments. All shrimp experiments in the present study were conducted in accordance with the guidelines of the Animal Research and Ethics Committees of the South China Sea Institute of Oceanology, Chinese Academy of Sciences.

### Cross-genomic, phylogenetic and conservation analysis of *Lv-Amy*

A total of 29 representative species were selected from GenBank for comparative genomic analysis (S1 Table). Candidate *Amy* genes were identified and enumerated based on functional annotations in the SwissProt database by using BLAST 2.9.0+ (-p blastp -e 1e-5). The species phylogenetic framework was obtained from LifeMap (https://lifemap.cnrs.fr/) and visualized and annotated using Chiplot (https://www.chiplot.online/) [18].

Amy protein sequences were aligned using the MUSCLE algorithm in MEGA v6.0 with default parameters and a neighbor-joining (NJ) phylogenetic tree was constructed under the Poisson substitution model with 1,000 bootstrap replicates to assess branch support. Sequence conservation of Amy proteins was evaluated in R v4.3.2 by generating sequence logos with the ggseqlogo package, in which amino acids were color-coded according to their physicochemical properties.

### Structural analysis of Amy proteins

The domain organization of Amy proteins in *L. vannamei* was predicted using SMART (https://smart.embl.de/) with default parameters. The predicted domain structures were visualized as schematic diagrams in R v4.3.2 using the ggplot2 package, ensuring consistent scaling across proteins. The three-dimensional (3D) structural model of representative *Lv*-Amy protein was predicted using AlphaFold 3 (https://alphafoldserver.com/) [19] with default parameters.

### Molecular cloning of *LV-Amy* full-length cDNA

Total RNA was extracted from *L. vannamei* using TRIzol™ Reagent (Invitrogen, USA) and treated with DNase I (Invitrogen, USA) to remove genomic DNA. First-strand cDNA was synthesized using the PrimeScript™ II Kit (Takara, Japan). Based on the predicted *Lv-Amy* sequence from NCBI (XP_027225605.1), specific primers Amy-F and Amy-R (S2 Table) were designed for polymerase chain reaction (PCR) of the *Lv-Amy* open reading frame (ORF) with condition of 35 cycles of 94°C for 25 s, 62°C for 25 s, and 72°C for 30 s with a final extension at 72°C for 5 min. The amplification product products were ligated into the pMD™19-T vector (Takara, Japan), transformed into *DH5α* (JM109) and confirmed with three positive clones by Sanger sequencing.

### RNA sequencing and expression profiling analysis

Total RNA from the ontogenetic development stages of *L. vannamei* was extracted as described above. Illumina sequencing libraries were then constructed and sequenced on the Illumina NovaSeq 6000 platform to generate 150 bp paired-end reads. The RNA-seq data generated in this study have been deposited in the NCBI under the BioProject accession number PRJNA1332953. In addition, publicly available RNA-seq datasets of *L. vannamei* from various tissues (PRJNA713996) and from different molting stages (PRJNA288849) [20] were retrieved from the NCBI Sequence Read Archive (SRA) to complement our in-house data.

For data preprocessing, all three groups of datasets were subjected to quality control using fastp v0.23.2 to remove adapter sequences and low-quality bases. The resulting clean reads were then aligned to the *L. vannamei* reference genome (GCF_003789085.1) using HISAT2 v2.2.1 with default parameters. Gene expression levels were quantified as fragments per kilobase of transcript per million mapped reads (FPKM), and heatmaps of *Amy* gene expression patterns were generated using TBtools.

### Quantitative real-time PCR (qPCR) and fluorescence *in situ* hybridization (F*IS*H)

Total RNA was extracted as described above and used as a template for first-strand cDNA synthesis with Evo M-MLV RT Mix Kit with gDNA Clean (Accurate Biology, China). Specific primers qAmy-F and qAmy-R (S2 Table) were designed according to the obtained *Lv-Amy* cDNA sequence for qPCR. The qPCR was performed using SYBR Green Premix *Pro Taq* HS qPCR Kit (Accurate Biology, China) on Thermal Cycler Dice Real Time System III (TaKaRa, Japan). Relative expression levels were calculated using the 2^−∆∆Ct^ method with elongation factor 1-α (*EF1α*, GU136229.1) as the reference gene.

The hepatopancreas (Hp), stomach (St), stomach–midgut junction (SMJ, proximal midgut immediately distal to stomach), midgut (MG), and hindgut (HG) were selected as tissues for F*IS*H. Tissues were fixed in 4% paraformaldehyde at 4°C overnight, dehydrated through an ethanol series and embedded in paraffin. Sections were deparaffinized, rehydrated, and treated with proteinase K (20 µg/mL) for 10 min at 37°C. Digoxigenin (DIG)-labeled RNA probes specific for *Lv-Amy* (S2 Table) were hybridized to the sections overnight at 42°C. After stringent washing, the samples were incubated with murine anti-digoxin-labelled peroxidase and anti-DIG-biotin for 1h followed by four washes in PBS. HRP-labelled streptavidin diluted in 1:100 PBS was then applied for 15 min at room temperature. Signal amplification was performed using the TSA Fluorescence Kit and nuclei were counterstained with DAPI. Fluorescence signals were examined and imaged using a fluorescence microscope.

### Expression and purification of recombinant *Lv*-Amy protein

For efficient heterologous expression, a codon-optimized *Lv*-Amy sequence lacking the native signal peptide was synthesized (S1 Fig) and amplified by PCR using gene-specific primers (S3 Table). The PCR product was digested with *Nde*I and *Xho*I and inserted into the corresponding sites of the pET-28a expression vector, yielding a construct with a C-terminal His-tag. The recombinant plasmid was transformed into *E. coli* BL21(DE3) and colonies were selected on LB agar plates after recovery. Positive clones were confirmed by sequencing and expanded in LB medium. Cells were harvested by centrifugation, lysed by sonication and separated into soluble and insoluble fractions. The recombinant *Lv*-Amy protein was mainly localized in inclusion bodies which were solubilized and purified under denaturing conditions using Ni² ⁺ -NTA affinity chromatography. The purified protein was subsequently refolded by stepwise dialysis concentrated in PBS and analyzed by SDS-PAGE. Protein concentration was determined using the BCA assay.

### Activity assay of *Lv*-Amy

The activity of recombinant *Lv*-Amy protein was measured using an α-amylase assay kit (Nanjing Jiancheng, China) based on the starch–iodine colorimetric method. One unit (U) of activity was defined as the amount of enzyme required to hydrolyze 10 mg of starch in 30 min at 37°C and specific activity was expressed as U/mg protein. For the standard assay, 100 μL of enzyme solution was added into a reaction mixture containing 0.4 mg/mL soluble starch and incubated at 37°C for 7.5 min. The reaction was terminated by adding 500 μL of iodine reagent and 3 mL of double-distilled water, and absorbance was measured at 660 nm (A_660_) using a microplate spectrophotometer (BioTek, USA).

Based on preliminary experiments, the recombinant *Lv*-Amy protein was diluted to 100 μg/mL for activity assays. The optimal temperature assay was carried out by adding 100 μL of recombinant *Lv*-Amy protein into an equivalent reaction mixture and incubating at different temperatures (15, 20, 25, 30, 35, 40, 45, 55, 65, 75, 85, and 95°C) for 7.5 min. The optimal pH assay was carried out by adding 100 μL of recombinant *Lv*-Amy protein into an equivalent reaction mixture at different pH values (4.0, 4.5, 5.0, 5.5, 6.0, 6.5, 7.0, 7.5, 8.0, 8.5, 9.0 and 9.5, adjusted with glycine-NaOH, phosphate and citrate-phosphate buffers) and incubating at 37°C for 7.5 min. Subsequently, 500 μL of iodine reagent and 3 mL of double-distilled water were added and the absorbance at A₆₆₀ was measured using a microplate spectrophotometer (BioTek, USA). All measurements were performed in triplicate technical replicates (n = 3) for each condition, and data are presented as mean ± standard deviation (SD). Relative enzyme activity was calculated from the absorbance values, with the highest activity defined as 100% [21].

## Result

### Genomic distribution, phylogeny and conservation of *Amy* genes across species

**Fig 1:**
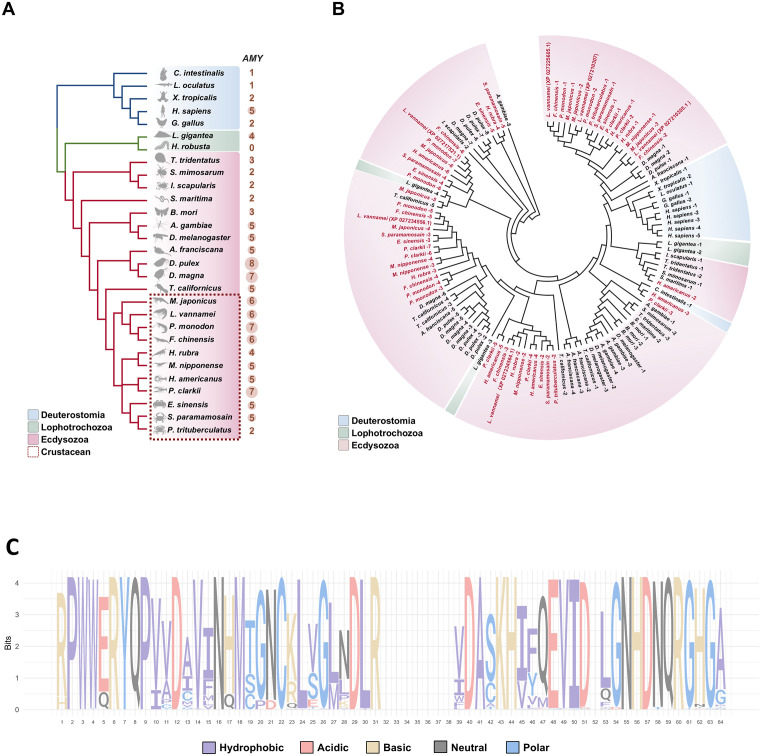
Genomic, phylogenetic and conservation analysis of *Amys* in 29 species. **A**: The number of *Amy* genes in 29 species across different taxa (Deuterostomia, Lophotrochozoa, and Ecdysozoa) with genomic data. **B**: Phylogenetic analysis of *Amy* genes from the 29 species using neighbor-joining method with 1000 bootstrap replicates. **C**: Sequence logo showing amino acid conservation patterns in Amy proteins, where letter height indicates relative residue frequency and amino acids are color-coded by physicochemical properties.

A cross-genomic and sequence conservation analysis was performed on 29 representative species covering Deuterostomia (Chordata), Ecdysozoa (Arthropoda) and Lophotrochozoa (Mollusca and Annelida), resulting in the identification of *Amy* genes for each species. Comparative analysis revealed that the number of *Amy* genes in Arthropoda showed an expansion trend, particularly in crustacean species (Fig 1A). The clustering of *Amy* was generally consistent with the evolutionary relationships between these species (Fig 1B). Multiple highly conserved amino acid residues were identified (Fig 1C), indicating that Amy has remained relatively conserved during evolution. Furthermore, the six *Amy* genes could be classified into three categories based on the branching patterns of the phylogenetic tree (Fig 1B).

### Domain architecture and expression profile of *Amy* in *L. vannamei*

**Fig 2:**
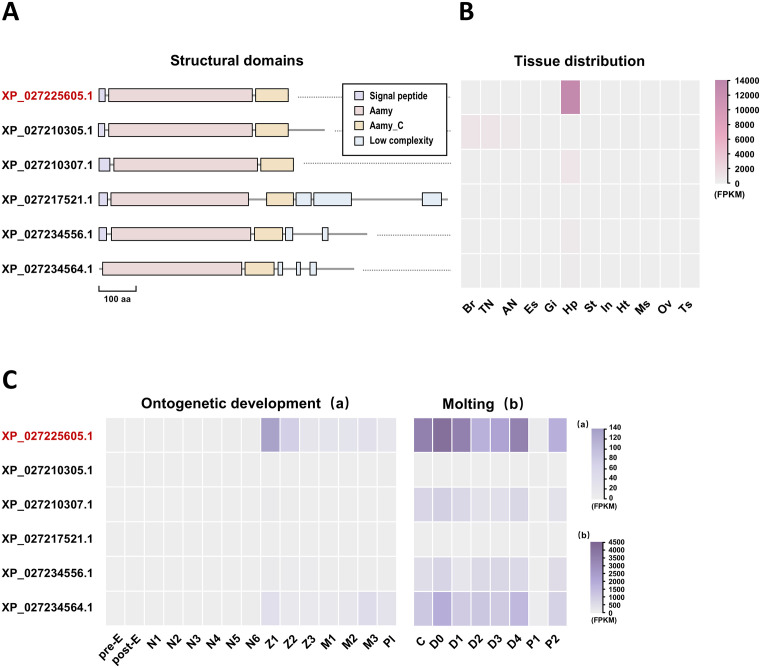
Domain organization and transcriptomic expression patterns of the *Amy* family in *L. vannamei.* **A**: The structural domain organization of Amy proteins in *L. vannamei* annotated by SMART. **B**: Transcriptomic expression in different tissues (n = 3 biological replicates). Tissues: brain (Br), thoracic nerve (TN), abdominal nerve (AN), eyestalk (Es), gill (Gi), hepatopancreas (Hp), stomach (St), intestine (In), heart (Ht), muscle (Ms), ovary (Ov), testis (Ts). **C**: Transcriptomic expression in different ontogenetic development and molting stages (n = 3 biological replicates). Ontogenetic development stages **(a)**: early fertilized egg (pre-E), late fertilized egg (post-E), nauplius stages (N1-N6), zoea stages (Z1-Z3), mysis stages (M1-M3) and postlarvae (Pl). Molting stages **(b)**: intermolt **(C)**, early to late pre-molt (D0-D4), post-molt (P1-P2).

Protein domain prediction of the Amy family in *L. vannamei* revealed that all members possess the typical α-amylase catalytic domain (Aamy) and a C-terminal domain (Aamy_C). Among them, five harbor a signal peptide, suggesting that they may be secreted via the classical secretory pathway to participate in the digestion of starch substrates and three contain a low complexity region (Fig 2A). In adult shrimp, tissue distribution analysis revealed that among the *Amy* family members, the transcript of XP_027225605.1 was highly and specifically expressed in the hepatopancreas, with an expression level at least 15-fold higher than that of any other *Amy* gene, while weak expression was detected in the intestine (Fig 2B). Furthermore, transcriptomic analysis was performed for two specific stages in *L. vannamei*. During ontogenetic development stage, all *Amy* genes were transcriptionally silent before the zoea stages and became partially activated upon entry into the zoea stages, while during molting, four *Amy* transcripts exhibited relatively high expression. Notably, XP_027225605.1 consistently displayed the highest expression across both stages (Fig 2C). Collectively, these findings indicate that XP_027225605.1 is the predominant *Amy* gene at the transcriptional level in *L. vannamei*.

### Molecular characterization and structural features of *Lv-Amy*

**Fig 3:**
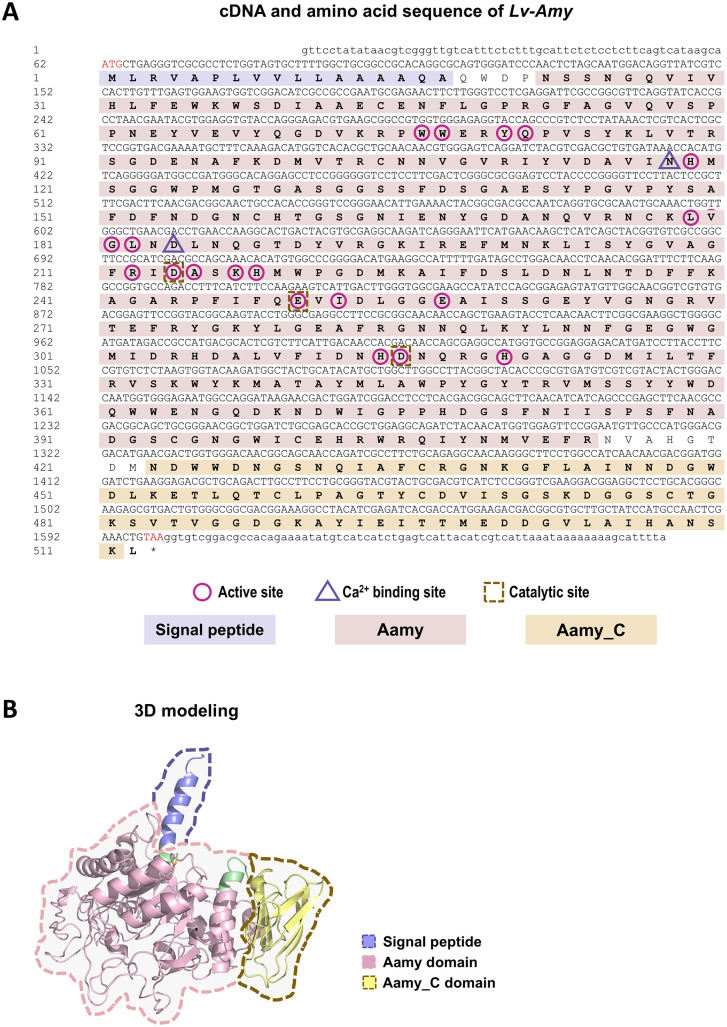
Full-length cDNA sequence and 3D structural modeling of *Lv-Amy.* **A**: Full-length cDNA sequence and deduced amino acid sequence of *Lv-Amy*. The translational start codon (atg) and stop codon (taa) are shown in red, the signal peptide, Aamy and Aamy_C are shown in the boxes with different colors, and the active site, Ca² ⁺ -binding site and catalytic site are indicated with different symbols. **B**: 3D structural model of *Lv*-Amy showing domain organization with the signal peptide, Aamy domain and Aamy_C domain distinguished by colors.

The cDNA sequence of *Lv-Amy* was obtained by PCR amplification. The *Lv-Amy* cDNA is 1673 bp in length, containing a 5’-untranslated region (UTR) of 61 bp, a 3’-UTR of 73 bp and an ORF of 1536 bp that encodes a 512 amino acids (a.a.) protein with a deduced molecular weight of 57.0 kDa and a predicted isoelectric point of 5.15 (Fig 3A). The *Lv-*Amy protein contains a signal peptide (Met 1 − Ala 17) at the N-terminal, followed by an Aamy domain containing the active site residues (Asn 27 − Arg 414) and two calcium-binding sites (Asn 118 and Asp 184) and an Aamy_C domain (Asn 423 − Leu 511) (Fig 3A). The 3D model was built up with the a.a. sequences of *Lv-Amy*. Base on the CaZy database prediction, *Lv-Amy* belongs to the glycoside hydrolase family GH13_24 and exhibits a classical (β/α)₈ TIM barrel fold (Fig 3B).

### Expression pattern and cellular localization of *Lv-Amy*

**Fig 4:**
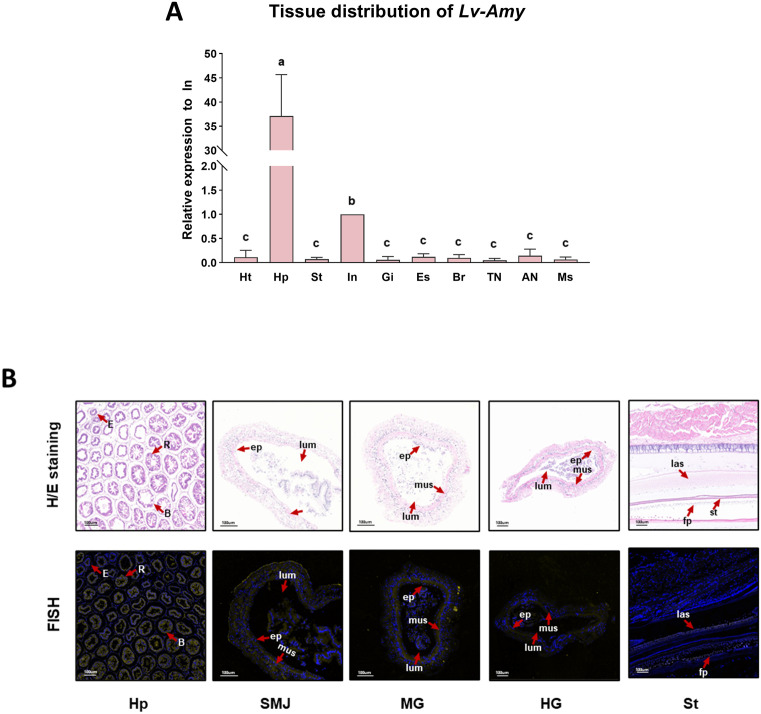
Tissue-specific expression and localization of *Lv-Amy.* **A**: Tissue-specific expression profiles of *Lv-Amy* detected by qPCR, with transcript levels normalized to the reference gene and expressed as mean ± SD (n = 3). Tissues follow abbreviations in Fig 2B. **B**: Histological and F*IS*H analysis of *Lv-Amy* in digestive tissues of *L. vannamei*. H/E stain indicates sections stained with hematoxylin and eosin. In the F*IS*H, yellow signals represent positive hybridization and blue signals indicate DAPI-stained nuclei. Tissues: hepatopancreas (Hp), stomach–midgut junction (SMJ), midgut (MG), hindgut (HG) and stomach (St). The hepatopancreas contains three major cell types: blister-like cells **(B)**, resorptive cells (R) and embryonic cells **(E)**. The intestine includes epithelium (ep), muscle (mus) and lumen (lum). The stomach contains lower ampullary setae (las), food particles (fp) and setules (st).

To validate the transcriptomic data and identify the expression sites of *Lv-Amy* in digestion-related tissues, tissue-specific expression profiles were analyzed in *L. vannamei* using qPCR and F*IS*H. The qPCR results were consistent with the trends observed in the transcriptome, with *Lv-Amy* significantly highly expressed in the hepatopancreas and relatively highly expressed in the intestine (Fig 4A). F*IS*H analysis further revealed the localization of cells expressing *Lv-Amy* mRNA in the hepatopancreas, intestine, and stomach, the main digestive organs of *L. vannamei*. To further investigate the expression patterns of *Lv-Amy* in different regions of the intestine, the intestine was divided into stomach–midgut junction, midgut, and hindgut for separate analysis. In the hepatopancreas, fluorescence signals were detected in blister-like cells, resorptive cells, and embryonic cells. In the intestine, signals were predominantly confined to the epithelial cells with similar expression patterns observed in the stomach–midgut junction, midgut, and hindgut. In the stomach, fluorescence was exclusively localized to the lower ampullary setae (Fig 4B). The F*IS*H results aligned with the qPCR, confirming the concordance between spatial localization and quantitative expression patterns.

### Expression and purification of recombinant *Lv*-Amy protein

**Fig 5:**
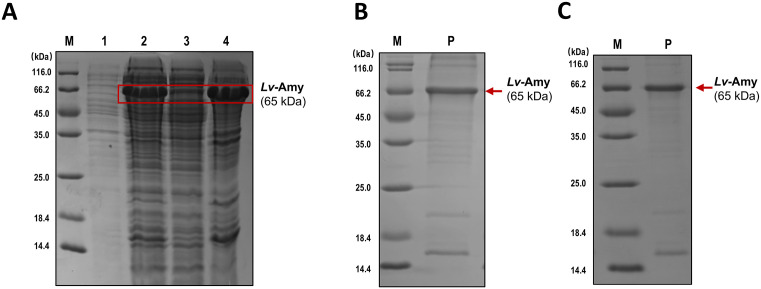
Expression, purification and refolding of recombinant *Lv*-Amy protein. **A**: SDS-PAGE analysis of whole-cell proteins, soluble fraction and insoluble fraction from IPTG-induced *E. coli* expressing *Lv*-Amy. **B**: SDS-PAGE of inclusion body proteins purified under denaturing conditions by Ni² ⁺ -IMAC. **C**: SDS-PAGE of refolded protein. M: Protein molecular weight markers, 1: control, 2: whole-cell proteins after IPTG induction, 3: soluble fraction after cell lysis, 4: insoluble fraction after cell lysis, P: target protein (red arrow).

For further characterization of the biological activities of *Lv-Amy*, a codon-optimized cDNA sequence was synthesized and expressed in *E. coli* as a C-terminal His-tag fusion. The recombinant protein was purified using immobilized metal-ion affinity chromatography (IMAC). Following Isopropyl-beta-D-thiogalactopyranoside (IPTG) induction, SDS-PAGE analysis revealed a distinct band at approximately 65 kDa (including ~4 kDa from the His-tag), consistent with the predicted molecular weight, indicating successful expression of the recombinant *Lv*-Amy protein (Fig 5A). Fractionation of the cell lysates showed that this ~65 kDa band was predominantly present in the insoluble fraction, suggesting accumulation in the form of inclusion bodies (Fig 5A). Under denaturing conditions, Ni² ⁺ -IMAC purification yielded enriched target protein bands (Fig 5B). Subsequent refolding generated soluble protein, and SDS-PAGE analysis after refolding displayed a prominent band at ~65 kDa, resulting in high-purity recombinant protein with a final concentration of 0.5 mg/mL (Fig 5C).

### Enzymatic properties and optimal catalytic conditions of *Lv*-Amy

**Fig 6:**
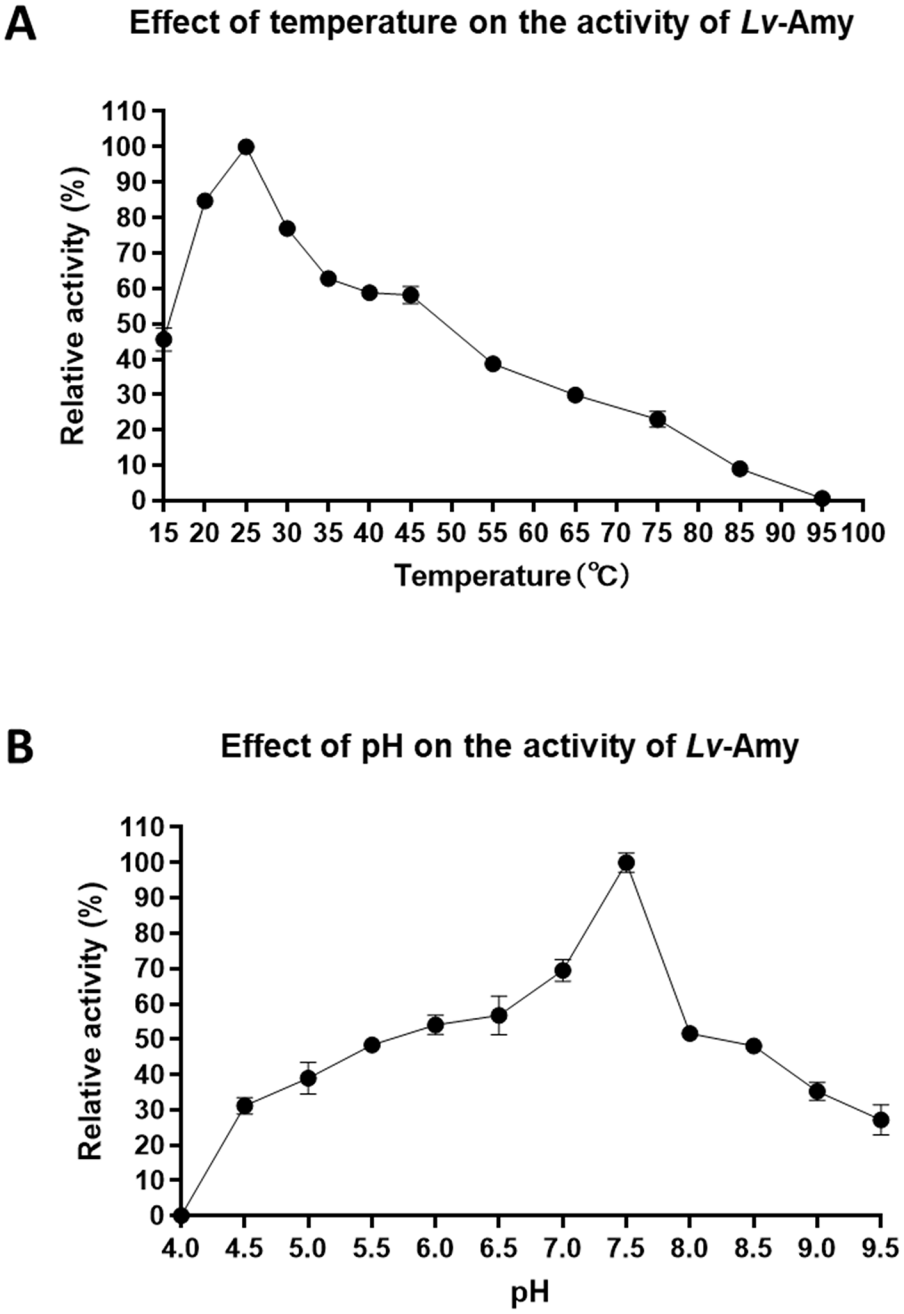
Effect of temperature and pH on the activity of recombinant *Lv*-Amy protein. **A**: Effect of temperature on the activity of *Lv*-Amy. **B**: Effect of pH on the activity of *Lv*-Amy. Values are shown as mean ± SD (n = 3 technical replicates per condition), error bars indicate SD.

To determine the optimal catalytic conditions of *Lv*-Amy, the enzymatic properties of the recombinant protein were assessed under a range of temperature and pH gradients. The results showed that *Lv*-Amy activity increased progressively with temperature, reaching its maximum at 25°C, and retaining more than 50% of its maximal activity within the range of 20–45°C. The activity declined sharply above 55°C and was completely lost at 95°C (Fig 6A). pH profiling revealed that *Lv*-Amy exhibited the highest catalytic activity at pH 7.5 and maintained over 50% of its maximal activity between pH 7.0 and 8.0. The enzyme was fully inactivated under strongly acidic conditions (pH 4.0), but retained more than 20% of its activity at pH 9.5, indicating relatively high tolerance to alkaline environments (Fig 6B). Taken together, these findings indicate that *Lv*-Amy is a low-temperature-adapted α-amylase with moderate alkali tolerance but limited thermal stability.

## Discussion

In this study, we comprehensively analyzed the α-amylase gene family in *L. vannamei* and across representative metazoan species, revealing an expansion of *Amy* copies in arthropods, particularly in decapods (Fig 1A). The clustering of *Amy* genes in the phylogenetic tree was largely consistent with the species’ evolutionary relationships (Fig 1B). Six *Amys* were identified in *L. vannamei*, and domain comparisons combined with transcriptomic analyses across multiple tissues, ontogenetic developmental stages and molting cycles (Fig 2) allowed us to define *Lv-Amy* (XP_027225605.1) as the predominant *Amy* gene at the transcriptional level. We further cloned its full-length cDNA, analyzed its tissue distribution and cellular localization, and successfully obtained recombinant protein in *E. coli*. Biochemical characterization of the purified recombinant *Lv*-Amy protein revealed optimal activity at pH 7.5 and 25°C (Fig 6).

Comparative genomic analyses revealed that, unlike the relatively stable gene copy numbers in deuterostomes and lophotrochozoans, the *Amy* gene family has experienced an expansion in arthropods, particularly in crustaceans (Fig 1A). Nevertheless, this expansion does not represent divergent evolutionary divergence traits, as Amy protein sequences remain highly conserved across species (Fig 1C). Such sequence conservation implies that catalytic activity is maintained under strong functional constraint, whereas the observed expansion can be attributed to ecological and dietary adaptations. However, digestive enzymes exhibit distinct features among animals with different feeding strategies [22]. For instance, CAZyme genes show evidence of positive selection in herbivorous crabs compared to their carnivorous counterparts [23], and multiple gene copies in insects potentially enhance dietary flexibility [24,25]. Similar phenomena have been observed in mammals: during domestication, dogs underwent a significant expansion of amylase gene copies to adapt to starch-rich diets [26]. Collectively, these findings suggest that *Amy* gene expansion and dietary adaptation represent convergent evolutionary patterns across diverse taxa. In *L. vannamei*, the presence of multiple *Amy* gene copies likely confers the capacity to optimize carbohydrate utilization under diverse ecological and nutritional contexts, consistent with its omnivorous feeding strategy. Based on these findings, we performed an in-depth analysis of six *Amy* genes in *L. vannamei* to investigate their potential functional differentiation and adaptive significance.

In *L. vannamei*, all six *Amy* copies contain both the characteristic Aamy domain and the Aamy_C domain but differ in other structural features such as the presence of signal peptides and low-complexity regions (Fig 2A). These structural differences are consistent with observations in insects [27], where *Amy* genes also maintain highly conserved catalytic domains but exhibit variability in intron number, N-terminal extensions and specific motifs [9]. This suggests that different *Amy* genes may have undergone functional differentiation with respect to secretion mode or enzymatic function. To further evaluate this hypothesis, we analyzed their expression patterns across different tissues, embryonic developmental stages, and molting cycles. In the adult shrimp of *L. vannamei*, *Amy* genes exhibit pronounced tissue specificity, with the highest expression levels detected in the hepatopancreas (Fig 2B). Notably, this high expression primarily originates from *Lv-Amy*, highlighting its central role as the predominant gene at the transcriptional level. During embryonic development, *Amy* genes transcription is scarcely detectable before the onset of exogenous feeding. However, once the larvae exhaust their yolk storage and reach the Z1 stage, *Amy* genes expression increased markedly, particularly that of *Lv-Amy* (Fig 2C). These findings are consistent with reports from other crustaceans: α-amylase activity intensifies markedly during the dietary shift from carnivory to omnivory in *Macrobrachium rosenbergii* larvae [28], while it continues to increase throughout development in *Maja brachydactyla* [29]. Collectively, these results indicate that α-amylase plays a pivotal role in establishing feeding capacity and mediating dietary transitions. In addition, multiple *Amy* transcripts in *L. vannamei* exhibit a pronounced upregulation during molting, peaking in the pre-molt stage, with *Lv-Amy* consistently showing the highest expression level (Fig 2C). Similar expression dynamics have been reported in *Panulirus argus* [30] and *Eriocheir sinensis* [31]. During the molting cycle, which is characterized by a sharp increase in energy demand, the global activation of *Amy* genes may represent an adaptive strategy to enhance carbohydrate degradation and glycogen storage, thereby supporting the energy requirements for tissue reconstruction and metabolic activity. In contrast to *Lv-Amy*, the other *Amy* genes generally exhibit much lower expression in the hepatopancreas. In particular, XP_027210305.1 shows detectable expression in neural tissues, including the brain, thoracic nerve and abdominal nerve, suggesting that some *Amy* genes may not be confined to the digestive system but may also participate in energy metabolism or signaling processes in the nervous system. Taken together, these expression patterns demonstrate that *Lv-Amy* functions not only as the primary *Amy* gene under stable physiological conditions but also as a key regulator of carbohydrate utilization and glycogen deposition during both the onset of juvenile feeding and the energy-intensive molting cycle. This highlights its crucial role in shrimp digestive physiology and developmental regulation.

Given its central role within the *Amy* gene family of *L. vannamei*, *Lv-Amy* was subjected to further investigation. qPCR results revealed that *Lv-Amy* exhibits significant tissue-specific distribution, with the highest expression in the hepatopancreas, followed by the intestine, and much lower in the stomach (Fig 4A). This expression pattern corresponds with the functional specialization of crustacean digestive organs: the hepatopancreas serves as the primary metabolic center for enzyme synthesis and secretion, the stomach facilitates mechanical grinding and primary digestion, and the intestine is mainly responsible for nutrient absorption [32–34]. Furthermore, F*IS*H at the cellular level further confirmed the expression characteristics of *Lv-Amy* (Fig 4B). Although the hepatopancreas appears to be the major source of *Lv-Amy*, the clearly detectable intestinal transcript levels, together with F*IS*H-positive signals in intestinal epithelial cells, suggest that part of the enzyme may also be synthesized in situ by the intestinal epithelium, in addition to being delivered from the hepatopancreas via the digestive fluid. These findings indicate a distinct spatial division of carbohydrate digestion in *L. vannamei*. First, food enters the stomach through the esophagus for mechanical processing. Subsequently, digestive enzymes such as *Lv-Amy* are synthesized and secreted by the hepatopancreas, and then transported to the intestine to hydrolyze carbohydrates including starch, ultimately providing substrates for absorption and utilization. This represents a coordinated system with clearly defined roles, ensuring efficient carbohydrate digestion and energy supply.

Concurrently, systematic analyses of *Lv*-Amy 3D structure and enzymatic properties were conducted. Structurally, *Lv*-Amy exhibits the classic (β/α)₈ TIM barrel conformation characteristic of α-amylases (Fig 3B). This structure consists of three main components: the catalytic core (β/α)₈ barrel domain A, the B domain extending beyond the barrel, and the C-terminal β-sheet domain C [8]. As a typical calcium-dependent metalloproteinase, α-amylase contains highly conserved calcium-binding sites across diverse biological phyla, including vertebrates [35], insects [36], and crustaceans [17]. In this study, two calcium-binding sites were identified in *Lv*-Amy, located at Asn and Asp residues, respectively (Fig 3A). The recombinant *Lv*-Amy protein exhibits α-amylase activity, with an optimal pH of 7.5 and an optimal temperature of 25°C. Because the native signal peptide was removed and *Lv*-Amy was produced in *E. coli* as a non-glycosylated protein, the biochemical parameters obtained here primarily describe the intrinsic catalytic core of it, whereas the properties of the mature secreted enzyme in vivo may be further modulated by post-translational processing and the digestive milieu. *Lv*-Amy maintains more than 50% of its maximum activity within the pH range of 7.0 to 8.0, indicating its pH adaptability (Fig 6B). Its optimal pH is consistent with those of α-amylases from the crustaceans *Penaeus californiensi*s [37] and *Portunus segnis* [38], and closely resembles the optimal pH of 7.0 reported for human pancreatic amylase [39]. Nevertheless, substantial interspecific variation exists in the pH preferences of α-amylases. Among crustaceans, while many enzymes function optimally under neutral to alkaline conditions, others are adapted to acidic environments, such as *P. argus* (pH 4.0–5.0) [40], *Homarus americanus* (pH 5.2) [41] and *Maguimithrax spinosissimus* (pH 5.5) [42]. In insects, the diversity is even greater, with optimal pH values ranging from acidic in *Diabrotica virgifera* (pH 5.7) [43], to neutral in *Scylla serrata* (pH 7.0) [44], alkaline in *Bombyx mori* (pH 9.2) [45], and up to strongly alkaline in *Naranga aenescens* (pH 10.0) [46]. Furthermore, the optimal temperature for α-amylase is also species-specific, though typically falling within the range of 35–45°C. For instance, human pancreatic amylase [47] and *Drosophila melanogaster* α-amylase [48] both show maximal activity at 37°C, whereas *N. aenescens* α-amylase peaks at 30°C [46]. By contrast, α-amylases from the crustaceans *P. segnis* [38] and *P. argus* (pH 4–5) [40] display maximal activity at 50°C. In this study, *Lv*-Amy maintained more than 50% of its maximum activity across the 20–45°C, with peak activity at 25°C (Fig 6A). This temperature range broadly overlaps the water temperatures experienced by *L. vannamei* in tropical marine and aquaculture environments, indicating that *Lv*-Amy can function efficiently under those conditions. However, it should be noted that these activity data were obtained using recombinant *Lv*-Amy expressed in a prokaryotic system, and the properties of the enzyme in vivo are likely to be further modulated by the digestive milieu and post-translational processing. Therefore, the present results cannot fully capture its precise optimal activity in vivo. In summary, *Lv*-Amy retains the highly conserved catalytic architecture characteristic of α-amylases, whereas its specific pH and temperature profiles reflect adaptive fine-tuning of its function to the digestive physiology of *L. vannamei* and its tropical marine environment.

## Conclusion

This study systematically characterized the composition, evolutionary relationships, expression patterns, and molecular characteristics of the *Amy* gene family in *L. vannamei*, with emphasis on its predominant gene *Lv-Amy* (XP_027225605.1). The results revealed that *Amy* genes are highly specifically expressed in the hepatopancreas and are markedly upregulated during periods of high energy demand, such as zoeal feeding and pre-molting, underscoring their central role in carbohydrate utilization and energy metabolism. Structural and enzymatic analyses further demonstrated that *Lv*-Amy retains the highly conserved catalytic structure of α-amylase, while the recombinant protein exhibits adaptive optimal pH and temperature responses to the tropical marine environmental conditions. By resolving developmental expression patterns of α-amylase and defining the enzymatic features of *Lv-Amy* in *L. vannamei*, this study provides theoretical insights into the molecular mechanisms of carbohydrate utilization in crustaceans and offers valuable references for aquaculture feed optimization.

## Supporting information

S1 Table The genome databases used in this study. (XLSX)

S2 Table Primers used in this study. (XLSX)

S3 Table List of species abbreviations used in this study. (XLSX)

S1 Fig Codon alignment of the original and optimized *Lv-Amy* ORFs. ORF sequence alignment before and after codon optimization (excluding signal peptides). Changed nucleotides and amino acids are highlighted in red.
(TIF)

S1 Raw images (TIF)
