# Supplementary figures and images for: Genome-wide identification and functional characterization of alpha-amylase genes in Litopenaeus vannamei
Source: PLoS One. 2026 Jan 13;21(1):e0338707. doi: 10.1371/journal.pone.0338707 (PMC12798967; doi:10.1371/journal.pone.0338707)

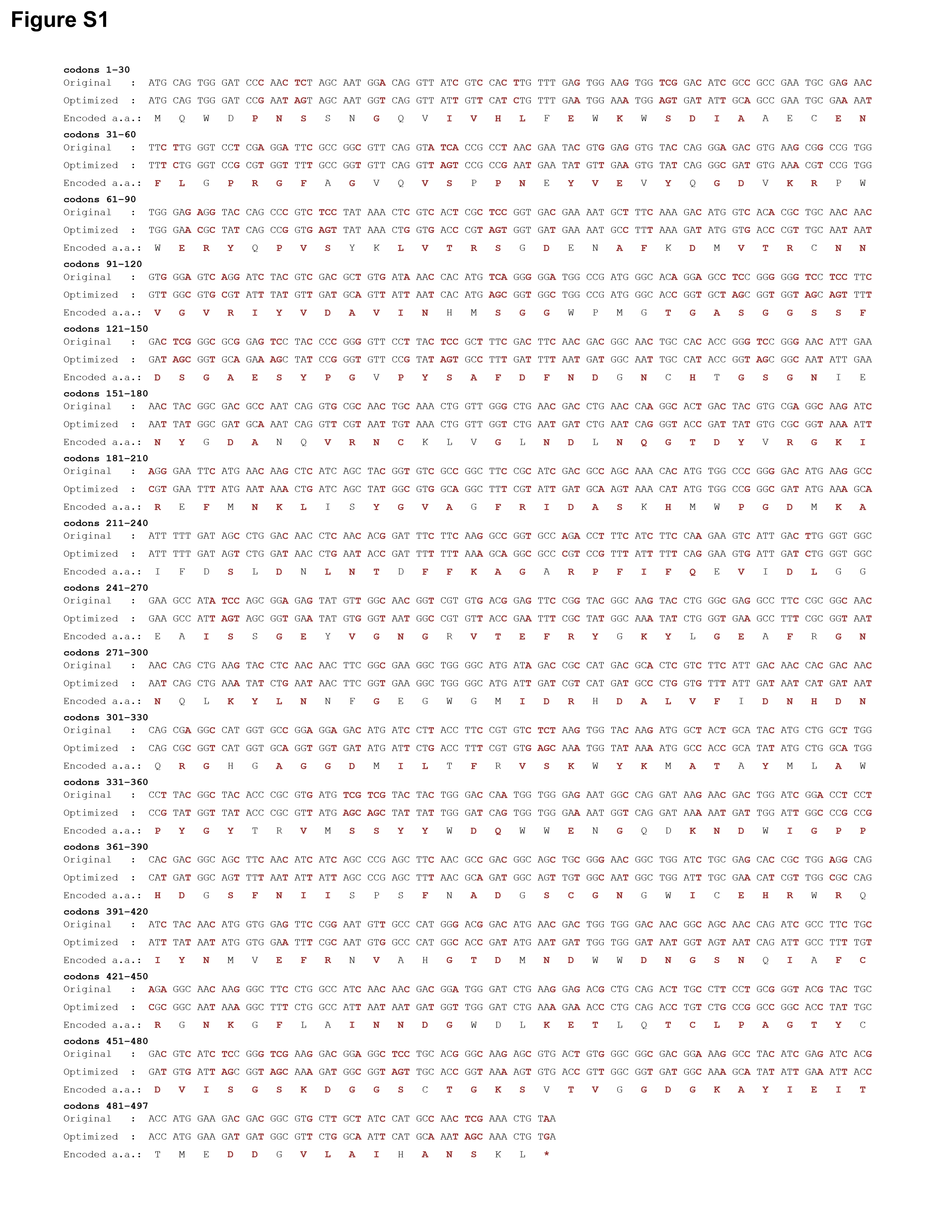

Supplement: S1 Fig — ORF sequence alignment before and after codon optimization (excluding signal peptides). Changed nucleotides and amino acids are highlighted in red. (TIF) [file pone.0338707.s004.tif]

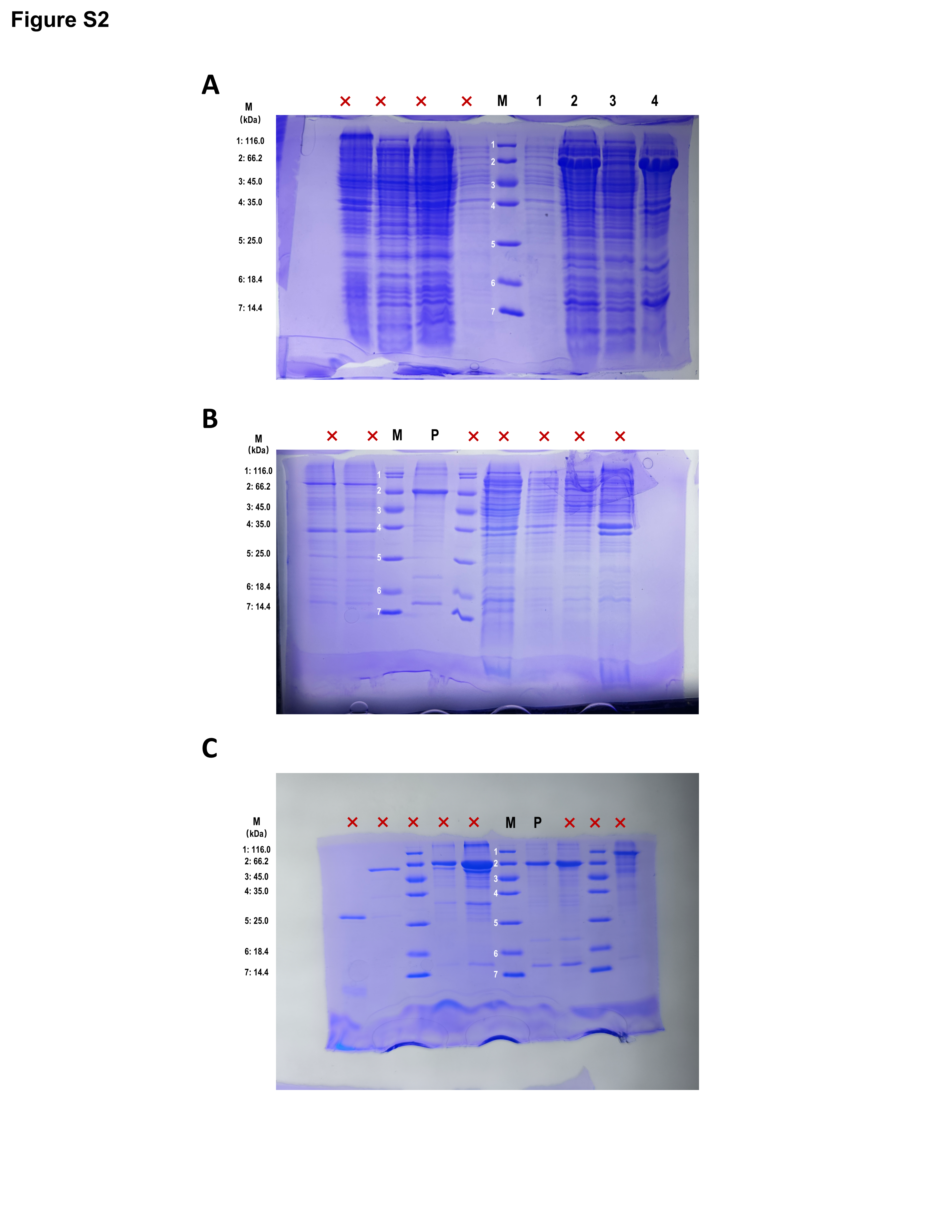

Supplement: S1 Raw images — (TIF) [file pone.0338707.s005.tif]
